# Reassessing Social Connectedness: Empirical Evaluation of WHO’s Theoretical Model and a Model based on Social Capital

**DOI:** 10.21203/rs.3.rs-10705575/v1

**Published:** 2026-09-09

**Authors:** Ester Villalonga-Olives, Zonggui Li, Ichiro Kawachi, Yuxi Shi, Sunmin Lee

**Affiliations:** University of Maryland, Baltimore; University of Maryland, Baltimore; Harvard University; University of California, Irvine; University of California, Irvine

**Keywords:** social connectedness, social capital, measurement model, confirmatory factor analysis, Asian American

## Abstract

**Background:**

Social connectedness is a key social determinant of health, influencing morbidity, mortality, and well-being. The World Health Organization (WHO) proposed a framework defining social connectedness across three dimensions: structure, function, and quality. This framework operates at a higher conceptual level than traditional social capital approaches, which emphasize social ties and access to resources. Empirical comparisons of these frameworks remain limited. This study examines whether the WHO framework is supported by empirical data in a sample of Asian Americans and compares it with established social capital-based dimensions. Given Asian Americans’ distinct histories of immigration, discrimination, cultural norms, and the limited existing research, this study provides a foundation for measuring social connectedness in this population.

**Methods:**

Data were drawn from 779 Asian American adults participating in the first wave of the DREAMS study (2022–2026). Survey items were mapped onto two frameworks: (1) a social capital-based model including social strain, social support, bridging and bonding social connections, and neighborhood social cohesion; and (2) the WHO social connectedness model including quality, functional, and structural dimensions. Confirmatory factor analysis (CFA) was applied to evaluate measurement models. Indicator selection was based on statistical performance and conceptual alignment. Model fit was assessed using CFI, TLI, RMSEA, and SRMR.

**Results:**

Both frameworks demonstrated good model fit after removing poorly performing indicators. In the social capital-based model, four dimensions were retained with moderate to strong factor loadings and good fit (CFI = .991; RMSEA = .058). In the WHO model, three dimensions were retained, with strong loadings and good fit (CFI = .990; RMSEA = .071). Across both frameworks, latent factor correlations were small, indicating distinct but related dimensions. Higher-order and bi-factor models did not converge, supporting a multidimensional structure.

**Conclusions:**

Findings support both the WHO and social capital frameworks as multidimensional representations of social connectedness. The choice of framework should be guided by research objectives: social capital models may be preferable for examining inequality and resource-based mechanisms, whereas the WHO model may be advantageous for population monitoring and intervention design. Preserving dimensional specificity can strengthen measurement and improve understanding of how social connectedness influences health outcomes.

## Background

Social connectedness has been defined as the ways in which people relate to and interact with one another (1). It is a social determinant of health that benefits societies broadly by improving population well-being, educational outcomes, resilience, and economic productivity (2-5). The World Health Organization (WHO) identified priority areas for research on social connectedness including identifying vulnerable groups, understanding mechanisms that link social ties to health, and designing interventions and policies capable of fostering healthier, more connected communities worldwide (1). Studying social connectedness is essential because social disconnection is widespread and has profound health impacts. The 2025 report of the WHO Commission on Social Connection reveals that one in six people worldwide experiences loneliness, a condition associated with more than 871,000 deaths each year—equivalent to roughly 100 deaths every hour (1, 6). Social isolation affects up to one-third of older adults and one-quarter of adolescents, underscoring that disconnection spans age groups and regions (1, 6). Loneliness and social isolation increase risks for cardiovascular disease, diabetes, cognitive decline, depression, anxiety, and premature mortality, while strong social connections offer protective effects and support healthier, longer lives (1, 7).

Social connections are commonly conceptualized as sources of social capital, defined as the resources that individuals and groups can access through their social relationships (8, 9). These resources include tangible forms of assistance—such as instrumental, emotional, or informational support—and other assets such as trust and shared norms of cooperation. Social capital can take two forms. First, at the individual level, social capital manifests when people draw on their personal networks for support and reflects the resources embedded in personal networks. Second, at the collective level, social capital manifests when shared norms and trust facilitate mutual aid and coordinated action within a group. Shared trust and norms of mutual assistance enable groups to function effectively. Together, these concepts clarify how social connections operate across levels and provide a foundation for understanding how social environments shape population health (8, 10-13).

The WHO Commission on Social Connection outlined a new theoretical framework in which social connectedness emerges from the interplay of three key dimensions: structural, functional, and quality dimensions (1). The structural dimension captures the objective aspects of social life, such as the number of relationships, the frequency of contact, and the presence or absence of roles and interactions; social isolation represents a deficit in this structural domain. The functional dimension refers to the types of support—emotional, practical, instrumental, or informational—that individuals receive from their networks. The quality dimension concerns how people evaluate their relationships, including whether they view them as positive or negative, close or distant. Loneliness, in this framework, reflects the subjective experience of a gap between desired and actual relational quality or quantity. Taken together, these dimensions provide WHO’s foundation for understanding how social connectedness protects or undermines health. The WHO framework is agnostic about network type (e.g. bonding vs bridging social connections), as it focuses more on form, function and experience and less on social position or network composition.

The WHO framework could be observed as operating at a relatively high level, whereas the more traditional dimensions derived from social capital theory were developed in the context of scholarship on inequality, power, and access to resources and, importantly, distinguish between the type and composition of social ties as well as the resources embedded within them (bonding vs bridging ties).

While the WHO framework does not replace social capital theory, it offers an additional way of conceptualizing social connections. Because the WHO framework operates at a higher level, there is some conceptual overlap, and indicators of social connectedness can be mapped onto both frameworks. Our objective is to examine whether the WHO framework of social connectedness is supported by empirical data and to compare it with established dimensions of social connections derived from social capital theory. We are interested in studying social connectedness in Asian Americans, a population that has been understudied. Ultimately, this work aims to inform the development of optimal measurement models and the construction of scores for future analyses examining the relationship between social connectedness and health outcomes in this population.

We test the two models of social connectedness in a sample of Asian Americans for several reasons. First, Asian Americans include a large proportion of immigrants who may be particularly vulnerable to experiencing a deficit of social connections, particularly bridging social connections vs. bonding social connections, due to segmented assimilation. Portes and Zhou, in their Segmented Assimilation Theory, explained how discrimination not only blocks the progress of assimilation but actively channels, or “pushes,” certain immigrant groups into the margins of society (14). Experiences of discrimination among Asian Americans date back to the Chinese Exclusion Act of 1882, and have continued to the present, as evidenced by attacks on Asian Americans during the COVID pandemic. Furthermore, limited English proficiency among many Asian American subgroups may serve as an additional barrier to assimilation. Therefore, immigrants may be cut off from mainstream social connections and opportunities. We are interested in examining the interplay between various constructs of social connectedness as well as understanding which constructs are more salient among Asian Americans. Second, Asian American groups are particularly interesting to study because of cultural characteristics (e.g., collectivist orientation and strong filial piety as an expression of Confucianism), which make negative aspects of social connection more salient. Third, in order to better understand social connectedness within this understudied population, there is a pressing need to develop validated instruments that capture unique aspects of Asian Americans’ social connectedness (e.g., potential deficits in bridging social connections). Testing the two models of social connectedness will provide measurement models to initiate the investigation of social connectedness among Asian Americans.

## Methods

### Procedure

Data were drawn from the Disparities Research to Evaluate Asians’ Multidimensional Stress on Sleep and Health (DREAMS) Study, which examines stress, resilience, sleep health, and related cardiometabolic disease risks among 779 Chinese, Korean, and Vietnamese adults. Bilingual research staff recruited participants aged 30 years and older from Greater Los Angeles and Orange Counties, California. Recruitment was conducted through community organizations, flyers, ethnic media, social media, community events, religious centers, and personal networks. Eligible participants provided informed consent and completed the survey in English, Cantonese, Mandarin, Korean, or Vietnamese. The survey was translated by bilingual staff and reviewed by a second bilingual staff. Then, it was pilot-tested by representatives from each ethnic community. Baseline data were collected from August 2022 to July 2024. Researchers regularly reviewed recruitment data to ensure the sample closely aligned with Census data on Chinese-, Korean-, and Vietnamese Americans in Orange and LA counties with respect to age, sex, education, income, and nativity. The study was approved by the institutional review board of the University of California, Irvine.

### Sample

The analytic sample comprised 779 Asian American adults drawn from the baseline DREAMS study. These data were used to develop and validate measurement models of social connectedness for Asian American populations who live in the United States. No missing data were reported in the baseline data. Participants had a mean age of 55.32 years old (SD = 15.42; range = 29.99–91.89), with 345 males and 434 females. Additional demographic information is summarized in Table 1.

### Statistical Analysis

#### Mapping Items to Theoretical Models

In this manuscript, the term framework is used to denote conceptual organization, while model is reserved for the operationalization and empirical estimation of the performance of the indicators. Indicator to model mapping assignments is presented in Table 2, which summarizes the indicators assigned to each dimension within the two frameworks and the corresponding concepts evaluated to support the analyses presented in this study. Detailed information on each indicator and response scheme is provided in supplementary material. In the first model we use a social capital-based model where social connectedness was conceptualized across five dimensions: Social Strain (SStr, 4 indicators); Social Support (SSpt, 7 indicators); Bridging and Bonding social connections (B&B, 5 indicators); Neighborhood Social Cohesion (NSC, 3 indicators); and Spiritual Social Connection (SpSC, 2 indicators). In the second model we use the WHO social connectedness model conceptualized across three dimensions: Quality of Social Connections (QSC, 6 indicators); Function of Social Connections (FSQ, 6 indicators); and Structure of Social Connections (SSC, 7 indicators). Please note that because indicators 6 and 7 conceptually aligned with both the QSC and FSC dimensions, they were included and tested under both dimensions.

#### Data Preparation

Two indicators, 22 (Number of situations with available support) and 23 (Number of social groups belonged to), were constructed as count variables. First, indicator 22 was recoded to represent the number of situations in which support was available, based on the sum of endorsed situations across items 22a – 22i, with range from 0 to 9. Second, indicator 23 was recoded to represent the number of social groups to which the participant belonged, based on the summed number of endorsed group categories, with range from 0 to 6. In addition, the raw count indicators 10 (Number of close friends) and 11 (Number of close relatives) exhibited substantial positive skew with maximum value as 100. To exclude outliers, both variables were winsorized at the 95th percentile, the maximum value for each indicator was reduced from 100 to 10. In order to place all the indicators on a common directional scale and to facilitate interpretation of the latent factors, the indicators for SStr were reverse coded so that higher values indicated stronger social strain. The supplementary material includes the full list of indicators used in this study, along with the survey questions, item numbers referenced in the manuscript, and descriptive information for each indicator (please see supplementary material).

#### Measurement model evaluation

Measurement model development proceeded using confirmatory factor analysis (CFA) in Mplus 8.11 via MplusAutomation in R (15, 16). Indicator selection was guided by statistical performance and conceptual interpretability: indicators with stronger loadings, greater explained variance ( *R*^2^), and clearer alignment with the intended construct were retained. Although no single cutoff was applied, loadings around or above .50 were generally preferred. Models were estimated using the weighted least squares mean- and variance-adjusted (WLSMV) estimator, which is suitable for ordinal indicators. Besides factor loadings, *R*^2^ values, conceptually alignment, model evaluation also depends on global model fit indices with commonly used guidelines: Comparative fit index (CFI) and Tucker-Lewis index (TLI) values ≥ .95, Root mean square error of approximation (RMSEA) ≤ .06, and Standardized root mean square residual (SRMR) ≤ .08 are interpreted as evidence of good fit (17).

Measurement model evaluation proceeded sequentially. First, first-order factors were evaluated separately within each dimension to identify poorly performing indicators. Second, retained indicators were re-estimated in refined individual-factor models. Third, the refined factors were combined into first-order correlated models to evaluate the overall measurement structure for each framework. Finally, higher-order and bi-factor models were tested for each framework. Detailed indicator-level decisions are reported in Table 3.

## Results

### Social capital-based measurement model

#### Model evaluation and refinement

Under the social capital-based model, five candidate dimensions were evaluated: SStr, SSpt, B&B, NSC, and SpSC. In the first step of analysis, each hypothesized factor in the traditional social capital-based framework was evaluated separately. SStr was retained without indicator deletion, as their indicators showed strong standardized loadings (.795 – .887) and substantial explained variance ( *R*^2^ = .632 – .787). Similarly, B&B was retained without indicator deletion, with standardized loadings ranging from .671 to .973 and explained variance from .450 to .946. Both SStr and B&B have relatively good model fit in the individual-factor models. SSpt was initially tested with seven indicators; however, indicators 10 (Number of close friends) and 11 (Number of close relatives) showed weak loadings (.232 and .426, respectively) and low explained variance (.054 and .182, respectively) and were therefore removed. The final retained SSpt indicators showed strong standardized loadings ranging from .754 to .922, with *R*^2^ values from .568 to .850. NSC was initially tested with five indicators, and indicators 15 (Feel safe in neighborhood) and 16 (Neighbors are friendly) showed very weak loadings (.098 and .074, respectively) and low explained variance (.010 and .005, respectively). To avoid just the identified model, we decided to drop one indicator, and indicator 15 (Feeling safe in the neighborhood) was dropped because it is more conceptually distant from the latent construct than indicator 16 (Neighbors are friendly). The final retained NSC indicators showed standardized loadings of .703 to .922, with *R*^2^ values of .494 to .851. SpSC was not retained because only two indicators were available, which yield an under identified model. As a result, this dimension was removed from the latent factor model, though the indicators could still be used as observed covariates for future research. Therefore, the final retained dimensions were SStr, SSpt, B&B and NSC. Panel A in Table 3 provides the detailed decision process for these refinements.

The second step is to test the above individual models as an overall measurement model. The social capital-based model included all four first-order factors and all retained indicators. This model showed strong model fit, with CFI = .980, TLI = .976, RMSEA = .078, and SRMR = .056. Within this model, however, indicator 16 (Neighbors are friendly) again showed a very weak loading (.059), reflecting the conclusion from the initial factor-level analyses that it did not adequately represent the NSC factor. Accordingly, indicator 16 was removed, and the model was re-estimated. The refined first-order correlated model showed improved model fit, with CFI = .991, TLI = .988, RMSEA = .058, and SRMR = .032. Standardized loadings for the retained indicators remained moderate to strong across all four factors. The social capital-based model is presented in Fig. 1. Panel A in Table 4 presents the final set of retained indicators to each dimension, and their standardized factor loadings and *R*^2^. The model fit indices can be found in Table 5.

#### Factor correlations

Correlations among the latent factors in the social capital-based model were generally small in magnitude. Specifically, SStr was negatively correlated with SSpt (r = −0.215, p < .001), and positively correlated with NSC (r = 0.108, p = .007) and with B&B (r = .205, p < .001). SSpt was positively correlated with B&B (r = .240, p < .001) and showed a non-significant positive correlation with NSC (r = .067, p = 0.08). Finally, B&B was positively correlated with NSC (r = .217, p < .001). Overall, these correlation magnitudes suggest that the four dimensions capture related yet non-overlapped aspects of social capital.

#### Higher-order and bi-factor models

To evaluate whether the social capital-based model dimensions could be represented by a more general higher-order factor, the second-order and bi-factor models were also tested. Neither model converged. We therefore selected the first-order correlated measurement model as the final representation of the social capital-based framework.

### WHO social connectedness measurement model

#### Model evaluation and refinement

Under the WHO social connectedness framework, three candidate dimensions were evaluated: QSC, FSC, and SSC. The same two-steps process was applied to the WHO social connectedness framework. In the individual factor-level analyses, for SSC, seven candidate indicators were examined initially, only indicators 12 to 14 were retained because alternative indicators showed weak loadings (.136 – .329) and low *R*^2^ (.019 – .108). In the final SSC model, the retained indicators demonstrated standardized loadings of .676 to .968, with corresponding *R*^2^ values of .457 to .938, indicating moderate to strong indicator performance. For FSC, indicator 22 (Number of situations with available support) was removed because it showed relatively weak loading (.403) compared with the remaining indicators. The final retained FSC factor included five indicators, with standardized loadings ranging from .754 to .922 and *R*^2^ values ranging from .568 to .851. For QSC, indicators 6 (You can count on someone) and 7 (Someone loves you) were removed because they aligned better with the FSC dimension. Indicators 1 to 4 were retained as the final indicators for this factor, all of which showed strong standardized loadings ranging from .795 to .885 and *R*^2^ values from .631 to .783. Therefore, the final retained dimensions were SSC, FSC, and QSC. Panel B in Table 3 provides the detailed decision process for these refinements.

Next, the retained indicators were then combined into a first-order correlated model. The final merged model included factors SSC, FSC, and QSC. This model demonstrated good fit, with CFI = .990, TLI = .987, RMSEA = .071, and SRMR = .035. In the final merged model, standardized loadings ranged from .676 to .968 for SSC, .754 to .922 for FSC, and .795 to .885 for QSC. The corresponding *R*^2^ values ranged from .457 to .938, .568 to .851, and .631 to .783, respectively. The final WHO social connectedness model is shown in Fig. 2. Panel B in Table 4 presents the final set of retained indicators for each dimension, and their standardized factor loadings and *R*^2^. The model fit indices can be found in Table 5.

#### Factor correlations

Correlations among the latent factors in the WHO social connectedness model were also small. In the model shown in Fig. 2, SSC was positively correlated with FSC (r = .240, p < .001) and QSC (r = .204, p < .001), whereas FSC was negatively correlated with QSC (r = −0.215, p < .001). Because indicators under QSC reflected negative aspects of quality social connection, the negative correlation was expected. Overall, the WHO factors also appear related but distinct dimensions of social connectedness.

#### Higher-order and bi-factor models

Higher-order and bi-factor versions of the WHO model were also tested but did not converge. Given the small factor correlations, these results indicate that the WHO dimensions are better represented as correlated first-order constructs.

#### Summary of retained measurement structures

Across both conceptual frameworks, the empirical evidence favored a first-order correlated factor structure. In both the social capital-based model and the WHO social connectedness model, removal of weak indicators improved model fit and yielded more coherent and interpretable factors. However, the factor correlations were not strong enough to support stable higher-order or bi-factor models, and those alternative specifications failed to converge.

## Discussion

This study evaluated and compared two conceptual frameworks of social connectedness—testing the social capital-based model and the World Health Organization (WHO) social connectedness model—using a common set of indicators drawn from a large sample of Asian American adults. This study is one of the first to examine measures that comprehensively assessed social connectedness among Asian Americans. Overall, the results provide strong empirical support for both frameworks as multidimensional representations of social connectedness. Choosing one or the other will depend on the theoretical lens and research objectives guiding a study.

Across both models, confirmatory factor analyses supported correlated first-order measurement structures, with clearly interpretable and statistically robust dimensions. A key contribution of this work is the demonstration that both frameworks “hold” empirically when applied to the same underlying data. Our results suggest that they can be deployed strategically depending on study aims. For example, studies focused on social inequalities, access to resources, and power dynamics may benefit from the more structurally explicit distinctions emphasized in social capital theory, such as bonding versus bridging ties and neighborhood cohesion. In contrast, studies oriented toward intervention development, population monitoring, or cross-national comparability may find the WHO framework advantageous due to its emphasis on form, function, and subjective experience of social relationships.

The observed multidimensionality of social connectedness across both frameworks is particularly noteworthy. In neither framework did higher-order or bi-factor models converge. This finding aligns with prior theoretical work emphasizing that individuals may have frequent social contact without meaningful support, or strong emotional ties without broad social integration (18-20). This finding is important, as an overall score of social connectedness may conceal heterogeneity in how different dimensions relate to health outcomes. Another important contribution of this study lies in its implications for measurement development and validation, particularly for the WHO framework. Although the WHO social connectedness framework is relatively new and largely conceptual at present, our results provide empirical evidence that its core dimensions can be operationalized using existing survey items and yield strong psychometric performance.

Our study also contributes evidence specific to an Asian American population, a group that remains underrepresented in research on social connectedness and social capital. To the best of our knowledge, this study is one of the first to examine social connectedness measure among Asian Americans extensively. We included measures that capture unique and crucial aspects of social connection among Asian Americans, such as social strain and bonding and bridging of social connections. Findings demonstrated that the measure of bridging and bonding was positively correlated with not only social support but also with social strain. In our sample, bonding social connections were substantially more prevalent than bridging connections, a pattern commonly observed in immigrant populations, where social ties tend to be concentrated among family members and close-knit networks. Consistent with this pattern, more than 60% of participants reported limited engagement with individuals from different countries of origin or racial/ethnic backgrounds. We also found that individuals with a greater number of bridging connections reported higher levels of support, potentially reflecting the benefits of more diverse social ties. Notably, however, these relationships were also associated with higher levels of social strain, suggesting that bridging ties may be more demanding or challenging to maintain. Cultural values emphasizing collectivism and filial piety may also partially explain these patterns, as they can intensify obligations within close relationships and, in turn, increase social strain. Similar configurations characterized by stronger bonding than bridging ties have also been observed in our prior studies of Hispanic immigrant populations in the United States (21, 22).

Several limitations should be acknowledged. First, while the item pool was rich, certain domains—such as spiritual social connectedness(23, 24)—were represented by too few indicators to support latent factor modeling. Although these indicators were excluded from the measurement models, future studies should consider expanding item coverage to better capture these dimensions, particularly among Asian Americans, for whom spirituality is an important aspect of social life (25, 26). Second, the sample was drawn from a specific geographic region and consisted primarily of foreign-born Asian American adults, which may limit generalizability. Despite these limitations, the study has notable strengths. These include a well-characterized community-based sample, rigorous and transparent model-building procedures, and a direct empirical comparison of two influential conceptual frameworks using the same data. By providing empirically validated measurement models, this work advances the science of social connectedness measurement and demonstrates the applicability of the WHO framework across empirical studies. The retained measurement structures performed well in this sample. An important next step would be to test if our results are reproduced in other racial and ethnic groups, as well as in non-U.S. populations.

## Conclusion

In conclusion, our findings demonstrate that social connectedness is a multidimensional construct that can be meaningfully operationalized through multiple theoretical frameworks. Both the social capital and WHO social connectedness frameworks are supported empirically and offer distinct advantages depending on research goals. Preserving dimensional specificity will be essential for advancing theory, improving measurement, and clarifying the pathways through which social connectedness shapes health.

## Supplementary Material

This is a list of supplementary files associated with this preprint. Click to download.

• Supplementarydocument.docx

## Figures and Tables

**Figure 1:**
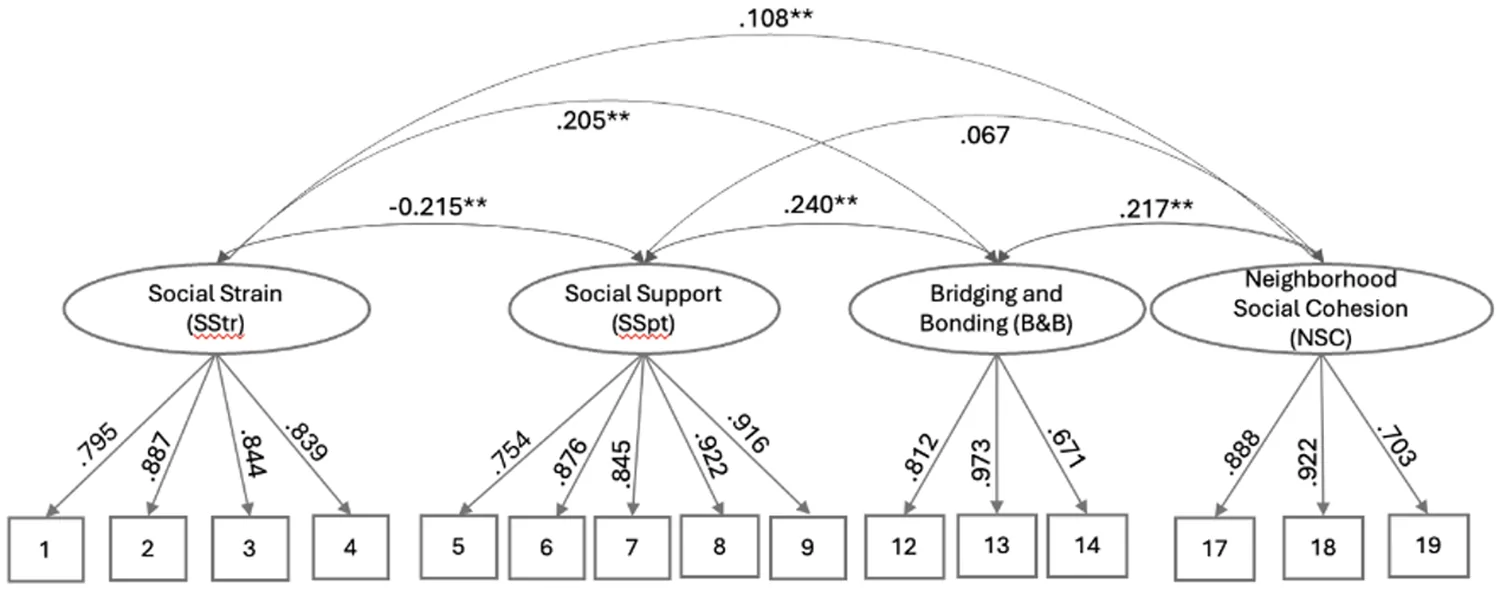
Framework A: Multidimensional social capital-based model Note. Model fit indices: CFI=.990, TLI=.987, RMSEA=.071, SRMR=.035. ** p < .01. Standardized parameter estimates are shown. Indicator residual variances were estimated but are omitted from the figure.

**Figure 2:**
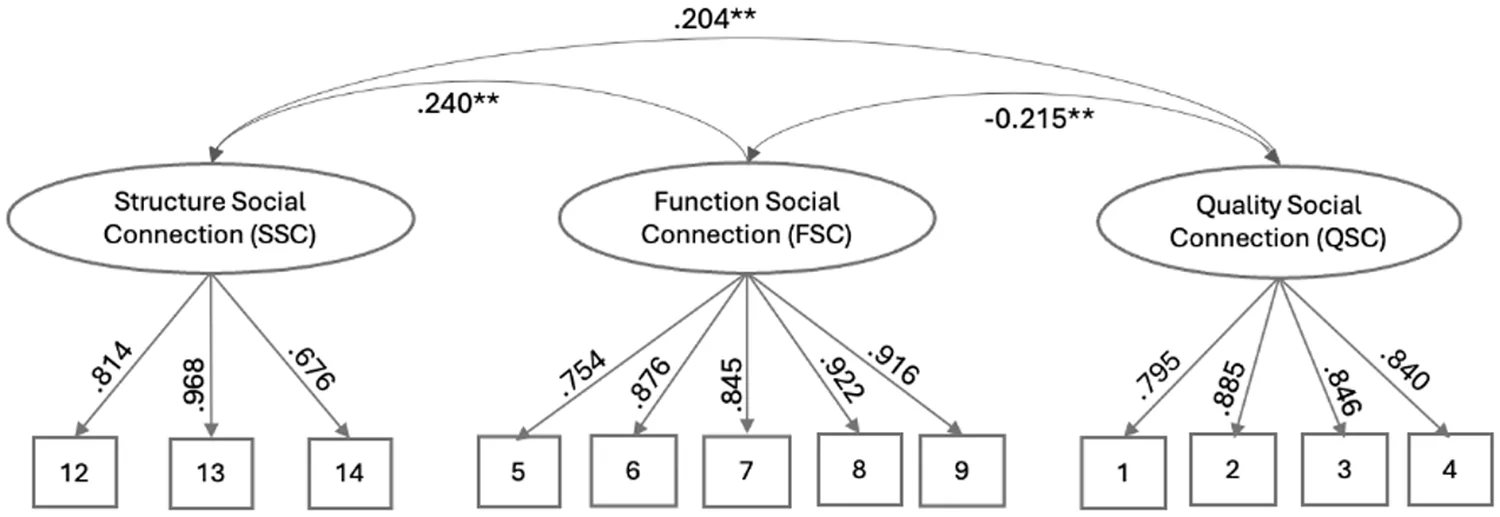
Framework B: World Health Organization (WHO) social connectedness model Note. Model fit indices: CFI=.99O, TLI=.987, RMSEA=.071, SRMR=.035. ** p < .01. Standardized parameter estimates are shown. Indicator residual variances were estimated but are omitted from the figure.

**Table 1:** Demographic statistics of participants

Total N	779	
Mean age (SD) and range	55.32 (15.42)	29.99–91.89
Variable	N	%
Gender		
Female	434	55.71
Male	345	44.29
Foreign-born status		
US-born	85	10.91
Foreign-born	694	89.09
Education		
Less than high school	72	9.24
High school graduate	130	16.69
Less than college graduate	130	16.69
College graduate	301	38.64
More than college	146	18.74
Employment		
Working full time	321	41.21
Working part time	108	13.86
Keeping house	73	9.37
Retired	200	25.67
Unemployed	77	9.88
Income		
< $20,000	148	19.00
$20,000-$49,999	169	21.69
$50,000-$99,999	131	16.82
> $100,000	219	28.11
Don’t know/Prefer not to say	112	14.38
Marital status		
Married/living as married	524	67.26
Divorced/separated/widowed	150	19.26
Never married	105	13.48
English proficiency		
Fluent/well	279	35.81
So-so	254	32.61
Poorly/Not at all	246	31.58

**Table 2:** Indicators mapped into each dimension under social capital-based model and WHO social connectedness model

Dimension	Indicator
1. Social capital-based model	
SStr: Social Strain	1: Family and friends demand you
	2: Family and friends criticize you
	3: Family and friends let you down
	4: Family and friends get on your nerves
SSpt: Social Support	5: Someone looks after you
	6: You can count on someone
	7: Someone loves you
	8: Someone gives you advice
	9: Someone gives you information
	10: Number of close friends
	11: Number of close relatives
B&B: Bridging and Bonding	12: Contact with people from different country
	13: Contact with people from different racial/ethnic background
	14: Contact with people from different level of education
NSC: Neighborhood Social Cohesion	15: Feel safe in neighborhood
	16: Neighbors are friendly
	17: Trust in neighbors
	18: Help from neighbors
	19: Attachment to neighborhood
SpSC: Spiritual Social Connection	20: Attend religious or spiritual services
	21: Seek comfort through religious or spiritual means
2. WHO social connectedness model	
QSC: Quality Social Connection	1: Family and friends demand you
	2: Family and friends criticize you
	3: Family and friends let you down
	4: Family and friends get on your nerves
	6: You can count on someone^1^
	7: Someone loves you
FSC: Function Social Connection	5: Someone looks after you
	6: You can count on someone^1^
	7: Someone loves you^1^
	8: Someone gives you advice
	9: Someone gives you information
	22: Number of situations with available support
SSC: Structure Social Connection	10: Number of close friends
	11: Number of close relatives
	12: Contact with people from different country
	13: Contact with people from different racial/ethnic background
	14: Contact with people from different level of education
	20: Attend religious or spiritual services
	23: Number of social groups belonged to

**Table 3:** Measurement model refinement across analytic steps

Dimension	Initial indicators	Initial model results	Refinement decision	Refined model results	Final decision
A. Social capital-based model					
SStr	1–4	Loadings ranged from .791 to .876; *R*^2^ ranged from .626 to .768; model fit: CFI = .995, TLI = .985, RMSEA = .126, SRMR = .015	No deletion	Retained as tested	Retained
SSpt	5–11	Indicator 10 and 11 showed weak loadings (.232 and .426) and low explained variance (*R*^2^ = .054 and .182); model fit: CFI = .887, TLI = .830, RMSEA = .429, SRMR = .090	Dropped 10 and 11	Stronger loadings for remaining Indicators (.752 – .923); model fit well	Retained
B&B	12–14	Loadings ranged from .808 to .910; *R*^2^ ranged from .652 to .828; model fit: CFI = .972, TLI = .915, RMSEA = .363, SRMR = .051	No deletion	Retained as tested	Retained
NSC	15–19	15 and 16 showed negligible loadings (.098 and .074) and minimal explained variance (*R*^2^ = .010 and .005); model fit: CFI = .963, TLI = .927, RMSEA = .230, SRMR = .068	Dropped 15	Stronger loadings across indicators 17–19 (.703 – .934)	Retained after further refinement
SpSC	20–21	Not retained due to model under-identified	-	-	-
First-order correlated model	1–9, 12–14, 16–19	Indicator 16 still showed weak loading (.059); model fit: CFI = .980, TLI = .976, RMSEA = .078, SRMR = .056	Dropped 16 from merged model	Moderate to strong loadings across all	Final retained model
Higher-order / bi-factor models	-	Not retained due to nonconvergence	-	-	-
B. WHO social connectedness model					
SSC	10–14, 20, 23	Indicators 10, 11, 20, and 23 showed weak performance, with loadings below .35 and low explained variance (*R*^2^ < .108); model fit: CFI = .852, TLI = .778, RMSEA = .271, SRMR = .107	Dropped 10, 11, 20, and 23	Stronger loadings for remaining Indicators (.670 – .973); model fit well	Retained
FSC	5–9, 22	22 showed relatively weak loading (.403) compared with other indicators	Dropped 22	Stronger loadings for remaining Indicators (.752 – .923); model fit well	Retained
QSC	1–4, 6–7	The broader QSC specification showed poor fit (CFI = .822, TLI = .703, RMSEA = .392, SRMR = .181); 6 and 7 were not retained here because they aligned better with the FSC dimension	Dropped 6–7	Stronger loadings for remaining Indicators (.791 – .854); model fit well	Retained
First-order correlated model	1–9, 12–14	Retained refined indicators from individual factor analyses were combined	No further item deletion	-	Final retained model
Higher-order / bi-factor models	-	Not retained due to nonconvergence	-	-	-

Note: SStr = Social Strain, SSpt = Social Support, B&B = Bridging and Bonding, NSC = Neighborhood Social Cohesion, SpSC=Spiritual Social Connection, SSC = Structure Social Connection, FSC = Function Social Connection, and QSC = Quality Social Connection. RMSEA was interpreted cautiously because small degrees of freedom can show inflated RMSEA even when the overall pattern of fit is strong.

**Table 4:** Standardized factor loadings and explained variance for the retained first-order correlated measurement models

Dimension	Indicator	Standardized factor loading	R-squared
A. Social capital-based model			
SStr	1: Family and friends demand you	.795	.632
	2: Family and friends criticize you	.887	.787
	3: Family and friends let you down	.844	.712
	4: Family and friends get on your nerves	.839	.705
SSpt	5: Someone looks after you	.754	.568
	6: You can count on someone	.876	.767
	7: Someone loves you	.845	.714
	8: Someone gives you advice	.922	.850
	9: Someone gives you information	.916	.839
B&B	12: Contact with people from different country	.812	.660
	13: Contact with people from different racial/ethnic background	.973	.946
	14: Contact with people from different level of education	.671	.450
NSC	17: Trust in neighbors	.888	.789
	18: Help from neighbors	.922	.851
	19: Attachment to neighborhood	.703	.494
B. WHO social connectedness model			
QSC	1: Family and friends demand you	.795	.631
	2: Family and friends criticize you	.885	.783
	3: Family and friends let you down	.846	.715
	4: Family and friends get on your nerves	.840	.706
FSC	5: Someone looks after you	.754	.568
	6: You can count on someone	.876	.767
	7: Someone loves you	.845	.714
	8: Someone gives you advice	.922	.851
	9: Someone gives you information	.916	.839
SSC	12: Contact with people from different country	.814	.663
	13: Contact with people from different racial/ethnic background	.968	.938
	14: Contact with people from different level of education	.676	.457

Note: SStr = Social Strain, SSpt = Social Support, B&B = Bridging and Bonding, NSC = Neighborhood Social Cohesion, SSC = Structure Social Connection, FSC = Function Social Connection, and QSC = Quality Social Connection.

**Table 5:** Fit indices for the retained first-order correlated measurement models

Framework	Chi-square (df)	CFI	TLI	RMSEA	SRMR
Social capital-based model	300.49 (84) **	.991	.988	.058	.032
WHO Social Connectedness model	253.21 (51) **	.990	.987	.071	.035

Note:

** indicates p≤ 0.001. RMSEA was interpreted cautiously because small degrees of freedom can show inflated RMSEA even when the overall pattern of fit is strong.

## Data Availability

The datasets used and/or analyzed during the current study are available from the corresponding author on reasonable request.

## Footnotes

1. Indicators 6 and 7 were mapped and tested under both the Quality Social Connection and Function Social Connection dimensions.

## Abbreviations

B&B: Bridging and bonding social connection
CFA: Confirmatory factor analysis
CFI: Comparative fit index
DREAMS: Disparities Research to Evaluate Asians’ Multidimensional Stress on Sleep and Health
FSC: Function of social connection
NSC: Neighborhood social cohesion
QSC: Quality of social connection
RMSEA: Root mean square error of approximation
SRMR: Standardized root mean square residual
SSC: Structure of social connection
SSpt: Social support
SStr: Social strain
SpSC: Spiritual social connection
TLI: Tucker-Lewis index
WHO: World Health Organization
WLSMV: Weighted least squares mean- and variance- adjusted
